# A Nontoxic Polypeptide Oligomer with a Fungicide Potency under Agricultural Conditions Which Is Equal or Greater than That of Their Chemical Counterparts

**DOI:** 10.1371/journal.pone.0122095

**Published:** 2015-04-07

**Authors:** Sara Monteiro, Alexandra Carreira, Regina Freitas, Ana Margarida Pinheiro, Ricardo Boavida Ferreira

**Affiliations:** 1 Centro de Botânica Aplicada à Agricultura, Instituto Superior de Agronomia, Universidade de Lisboa, Lisboa, Portugal; 2 Converde, SA, Biocant-Park, Cantanhede, Portugal; 3 Instituto de Tecnologia Química e Biológica, Universidade Nova de Lisboa, Oeiras, Portugal; University of California, UNITED STATES

## Abstract

There are literally hundreds of polypeptides described in the literature which exhibit fungicide activity. Tens of them have had attempted protection by patent applications but none, as far as we are aware, have found application under real agricultural conditions. The reasons behind may be multiple where the sensitivity to the Sun UV radiation can come in first place. Here we describe a multifunctional glyco-oligomer with 210 kDa which is mainly composed by a 20 kDa polypeptide termed Blad that has been previously shown to be a stable intermediary product of β-conglutin catabolism. This oligomer accumulates exclusively in the cotyledons of Lupinus species, between days 4 and 12 after the onset of germination. Blad-oligomer reveals a plethora of biochemical properties, like lectin and catalytic activities, which are not unusual per si, but are remarkable when found to coexist in the same protein molecule. With this vast range of chemical characteristics, antifungal activity arises almost as a natural consequence. The biological significance and potential technological applications of Blad-oligomer as a plant fungicide to agriculture, its uniqueness stems from being of polypeptidic in nature, and with efficacies which are either equal or greater than the top fungicides currently in the market are addressed.

## Introduction

Attempted infection of a plant by a fungal pathogen may be compared to a warfare whose major weapons are proteins derived from both plant and pathogen [1]. Plant resistance is determined by an impressive combination of both constitutive and inducible defence mechanisms that involve a vast array of proteins and other organic molecules produced prior to infection or during pathogen attack [2]. For example, the completion of the *Arabidopsis* genome sequence showed that this plant species has a few hundred open reading frames that encode potential surveillance proteins [3].

Antifungal proteins, as their name imply, serve a protective function against fungal invasion. They are involved in both constitutive and induced resistance to fungal attack and are produced by a multitude of organisms [4,5]. Hundreds of antifungal proteins have been described, with more being discovered almost daily. C. P. Selitrennikoff [6] considered thirteen families of antifungal proteins, which were named primarily on the basis of their mechanism of action (*e*.*g*. chitinases, β-glucanases), their structure (*e*.*g*. glycine rich), or their similarity to a known type of protein (*e*.*g*. thaumatin-like protein) [7]. Several proteins may be classified into more than one family and some are included in the group of pathogenesis-related (PR) proteins.

In plants, the chitin-binding proteins, one family of antifungal proteins, are usually divided in two classes: class I proteins contain a chitin-binding domain similar to a domain present in hevein, a protein from rubber latex [8]; class II proteins lack the chitin-binding hevein domain [7,8]. The antifungal activity of chitin-binding proteins is mainly due to their ability to bind fungal cell wall chitin, which results in disruption of cell polarity and consequent inhibition of growth by mechanisms that have not been elucidated [9]. The antifungal activity displayed by chitinases, another family of antifungal proteins, was originally assumed to derive from their ability to catalytically cleave chitin, leading to a weakened fungal cell wall and subsequent cell lysis. However, more recent evidence indicates that the mechanisms by which chitinases inhibit fungal growth seem to be more dependent on the presence of a chitin-binding domain than or chitinolytic activity [9].

Vicilins comprise a major group of legume seed storage globulins. Together with legumins, they usually account for approximately 80% of the total protein in mature legume seeds [10]. Vicilins are oligomeric proteins (150 to 170 kDa) with variable degrees of glycosylation, composed of three similar subunits of ~40 to 70 kDa, with no disulphide linkages and stabilized by non-covalent forces [10,11]. The combination of multiple structural genes and extensive post-translational processing, results in a high degree of subunit polymorphism for these proteins [12]. Nevertheless, vicilins from different legume seeds exhibit a considerable amount of sequence homology and similar 3-D structures [13–16]. Vicilins isolated from a variety of legume seeds have been reported to bind strongly to chitin, chitosan and fully acetylated chitin [17–19]. These interactions were proposed to explain their detrimental effects on the development of the cowpea weevil (*Callosobruchus maculatus*), a bruchid insect that is a pest of cowpea seeds [19,20] as well as their interference with the germination of spores or conidia of phytopathogenic fungi [21].

Recently, we described a new polypeptide, named Blad, a highly processed multifunctional product of *Lupinus* vicilin gene with a unique biosynthetic route [22]. Blad polypeptide is the major subunit of a glyco-oligomer, here termed Blad-oligomer, accumulates exclusively in the cotyledons of Lupinus species, between days 4 and 12 after the onset of germination. In this work, a considerable number of differential properties and physiological roles played by this remarkable oligomer at a very specific stage of *Lupinus* development were elucidated. A number of characteristics, deriving directly from the oligomer properties, highlight the potential technological significance of its application. In addition, the uniqueness of Blad-oligomer stems from being of polypeptidic in nature, edible and with efficacies which are either equal or greater than the chemical, usually highly toxic, top fungicides currently in the market.

## Materials and Methods

### Ethics statement

All necessary permits were obtained for the described field studies. All the field trials were performed in field trial lands owned by the research companies (Two bees Agric. Research, Synthec Research, FMC Ag. Products Group, Helena R&D, Crop Science and Pan Am. R&D) and specifically maintained for this purpose. In addition, the field studies did not involve endangered or protected species; they were performed with commercial available fruit varieties and with industrial-standards as controls.

The bee colonies were inspected periodically according to good bee keeping practice by an experienced apiarist. Furthermore, the colonies were examined for a reportable bee epidemic by an authorised bee specialist, without any negative findings.

### Biological material and growth conditions

Dry seeds of white lupin (*Lupinus albus* L.) cv. Leblanc were obtained from a local market. When appropriate, the seeds were germinated for periods up to 10 days [22]. In all cases, the seed coats were removed and the intact cotyledons dissected from the axes and stored frozen at −80°C until needed.

The majority of the fungal strains tested for MIC were purchased from reference culture collections (American Type Culture Collection—ATCC, and Centraalbureau voor Schimmelcultures—CBS): *Alternaria alternata* (CBS 154.31), *Cercospora zeae-maydis* (ATCC MYA-725), *Colletotrichum acutatum* (CBS 294.67), *Colletotrichum gloeosporioides* (CBS 119204), *Colletotrichum dematium* var. *trucatum* (ATCC 76264), *Colletotrichum graminicola* (CBS 130836), *Exserohilum turcicum* (ATCC 64836), *Fusarium graminearum* (CBS 184.32), *Fusarium oxysporum* (CBS 114750), *Macrophomina phaseolina* (CBS 205.47), *Mycosphaerella fijiensis* (CBS 116635 and 120258), *Sclerotinia sclerotiorum* (CBS 128069), *Verticilium dahlia*e (CBS 110277 and CBS 110275), *Verticilium alboatrum* (CBS 385.91). Two strains isolated in our lab were also used: *Botrytis cinerea* (isolated from tomato), and *Sclerotinia sclerotiorum* (isolated from lettuce). These strains were identified by sequence analysis of the internal transcribed spacer (ITS) region of the ribosomal DNA (PCR amplification with primers ITS1 and ITS4). For preparing the inocula, all fungi were grown on Sabouraud Dextrose Agar for 7 days at 25°C.

The honey bee test was carried out with young adult worker bees deriving from a healthy colony. One day before the start of the test, the bees were collected randomly from the outer combs of the colony for the oral and contact toxicity test. The hive used for the honeybee collection for the test was adequately fed, healthy and as far as possible disease-free and queen-right. During the experimental phase the test organisms were kept in constant darkness except at the start of the experimental phase in the oral toxicity test (feeding of the bees) and during the three assessments (4, 24 and 48 hours after test start). Feeding, application and assessments were made under neon light.

### Purification of proteins

Total globulins from *Lupinus* dry seeds were extracted and isolated as described by [22]. The globulins were subsequently precipitated by the addition of ammonium sulphate (561 g/L), centrifuged at 30,000 g for 20 min at 4°C, resuspended in 50 mM Tris-HCl buffer, pH 7.5 (5.7 mL/g of cotyledon) and desalted on PD-10 columns previously equilibrated in the same buffer. For the germinated seedlings, the cotyledons were ground and homogeneized with a mortar and pestle in water (pH adjusted to 8.0) containing 10 mM CaCl2 and 10 mM MgCl2 (2 mL/g fresh weight) incubated at 4°C for 30 min with agitation, filtered through cheesecloth and centrifuged at 30,000 g for 1 h at 4°C. The precipitate was suspended in the globulin solubilising buffer (2 mL/g fresh weight; 100 mM Tris-HCl buffer, pH 7.5, containing 10% (w/v) NaCl, 10 mM ethylenediaminetetraacetic acid (EDTA) and 10 mM ethyleneglycol-bis(β-aminoethylether)-*N*,*N*,*N'*,*N*'-tetraacetic acid (EGTA)) agitated during 30 min at 4°C and followed by a centrifugation step for 1 h at 30,000 g and 4°C and desalted on PD-10 columns. For individual globulins purification, the total fraction was fractionated and purified by AKTA anion exchange chromatography on a Q-Sepharose column (∅ = 1 cm; h = 8 cm; flow rate = 1.5 mL/min) essentially as described by [22]. The bound proteins were eluted with a gradient of NaCl (0 to 1 M) and desalted into 50 mM Tris-HCl buffer, pH 7.5. α- and γ-conglutins were purified from *L*. *albus* cotyledons as reported by [23].

Blad-oligomer was extracted and isolated from the cotyledons of eight-days old seedlings. The protein corresponding to β-conglutin was purified by AKTA anion exchange chromatography as explained above and subsequently subjected to AKTA gel filtration on the Superose 12 HR 10/30 column previously equilibrated in 50 mM Tris-HCl buffer, pH 7.5. This last purification step does not affect the polypeptide pattern of the oligomer, but removes unidentified low molecular mass compounds.

For the field trials studies, the germinated cotyledons were ground and homogeneized in a pilot scale hammer mill in water containing 10 mM EDTA and centrifuged at 9,000 *g* and 4°C. The supernatant was heated at 60°C during one hour and submitted to a new centrifugation step at the same conditions. Finally, the extract was concentrated in a speed-vacuum evaporator until it reaches 20% (w/v) of Blad-oligomer.

*Lemna* ribulose bisphosphate carboxylase was purified as described by [23], by a procedure involving extraction of the total soluble protein, AKTA anion exchange chromatography on the Mono Q HR 5/5 column, and AKTA gel filtration on the Superose 6 HR 10/30 column.

### Electrophoresis, immunoblotting and production of anti-Blad polyclonal antibodies

One-dimensional, sodium dodecyl sulphate-polyacrylamide gel electrophoresis (SDS-PAGE), western blotting and immunoblotting were performed as described before [24]. For anti-Blad polyclonal antibodies production, AKTA purified Blad-oligomer was subjected to preparative SDS-PAGE (10% w/v acrylamide). Total polypeptides were visualized with CuCl_2_ (negative staining; copper staining) [25]. Blad polypeptide was sliced and the protein eluted as described before [26], desalted on a PD-10 column previously equilibrated with water and utilized to immunize two-month-old, male Wistar rats. Each rat was injected subcutaneously with 0.4 mL of a solution containing 0.125 mg Blad and 0.2 ml complete Freund's adjuvant. To obtain a high titre, three identical booster injections were given every four weeks in complete Freund's diluted 1:10 with incomplete adjuvant. At intervals, blood was collected from the heart and the titre followed by immunoblotting. Total blood was taken from the heart 9 days after the third booster injection. Blood samples were allowed to clot and the serum was collected and stored frozen at -80°C. Antibody specificity was thoroughly assessed.

### Assays for protein phosphorylation and protein glycosylation

Detection of phosphoryl groups in proteins was performed according to the method described by [27]. After SDS-PAGE the proteins in gels were fixed overnight in 50% (v/v) methanol and 10% (v/v) acetic acid. The gel was washed in water and incubated with the fluorescent phosphosensor dye Pro-Q diamond (a phosphoprotein gel stain; Molecular Probes) during 2 h, under slow agitation, in the dark, and subsequently destained in 15% (v/v) propylenoglycol containing 50 mM sodium acetate, pH 4.0. The gel was finally analyzed in a fluorimeter Typhoon 8000 (Amersham; excitation λ = 532 nm; emission λ = 583 nm) and photographed. The same gel was then stained for total protein with Coomassie Brilliant Blue R-250.

Detection of carbohydrate residues in proteins was performed by affinoblotting. Proteins separated by SDS-PAGE and blotted onto a PVDF membrane were probed for the presence of glycopolypeptides essentially by the concanavalin A/peroxidase method developed by [28], as described before [29].

### Enzymatic assays

Blad-oligomer was assayed for the activities of β-*N*-acetyl-D-glucosaminidase, chitinase, chitosanase and endo-1,3-β-glucanase. Two hundred μg pure protein was used in each enzymatic assay. The controls used were: (i) substrate and buffer; (ii) purified proteins and buffer. All assays were made in triplicate.

β-*N*-Acetyl-D-glucosaminidase (EC 3.2.1.52) activity was measured essentially as reported by [30]. The reaction mixture consisted of 2.5 μmol of *p*-nitrophenyl-*N*-acetyl-β-D-glucosaminide, 10 μmol citric acid-sodium citrate, and 200 μg of the purified proteins in 1 mL of 10 mM sodium citrate buffer, pH 5.5. The amount of *p*-nitrophenol released was calculated from a standard curve (0.05 to 0.5 μmol *p*-nitrophenol). Enzyme activity is reported as pmol *p*-nitrophenol released per min per μg protein.

Chitinase (EC 3.2.1.14) activity was measured essentially by the method described by [30]. The chitinase assay is based on the colorimetric determination of *N*-acetylglucosamine released from chitin. The substrate for the reaction mixtures consisted of 300 mg finely ground chitin dispersed in 80 mL 0.2 M sodium citrate buffer, pH 5.5. The amount of *N*-acetylglucosamine released was determined from a calibration curve (0.68 to 4.5 μmol *N*-acetyl-D-glucosamine). Chitinase activity was expressed as pmol *N*-acetylglucosamine released per min per μg of protein.

Chitosanase (EC 3.2.1.132) activity was assayed as described by [31]. Chitosanase activity was determined by measuring the quantity of reducing sugars generated by hydrolysis of soluble chitosan. The substrate for this assay was chitosan dissolved in 50 mM sodium acetate buffer, pH 5.5. The reducing sugars were determined by reading the optical density at 405 nm, and comparison with a standard curve (0.05 μmol to 0.5 μmol D-glucosamine).

Endo-1,3-β-glucanase (EC 3.2.1.6) activity was quantified essentially by the method of [32]. Laminarin as the substrate and dinitrosalicylic reagent (150 mL 4.5% NaOH added to 440 mL solution containing 4.4 g 3,5-dinitrosalicylic acid and 127 g K-Na tartrate.6H_2_O) were used to measure the reducing sugars released. The amount of glucose released was determined by reading the optical density at 500 nm and the results calculated from a standard curve (0 to 11 μmol D-glucose). Glucanase activity was expressed in pmol glucose released per min per μg of protein.

### Fungal inhibition studies

#### Minimum inhibitory concentrations (MICs) determination

This susceptibility test was made according to the CLSI (former NCCLS) guideline M38-2A (Reference Method for Broth Dilution Antifungal Susceptibility Testing of Filamentous Fungi; CLSI) [33], using broth microdilution method, with small modifications The inoculum suspension was prepared by covering the colonies with approximately 5 mL of sterile 0.9% (w/v) saline (NaCl) with 0.01% (v/v) tween 20. The resulting suspension was transferred to a sterile tube, vortexed for 15 s, and the cell density adjusted to 0.4–5.0 x 10^6^ CFU/mL by direct counting of spores using a neubauer chamber. The final inoculum suspension was made by a 1:50 dilution with Potato Dextrose broth medium (pH 7.5), prepared with a double concentration, which resulted in a final concentration of approximately 0.4 x 10^4^ to 5 x 10^4^ cells per mL. The inoculum size was verified by enumeration of CFU obtained by subculturing on SDA plates. Filamentous fungi inocula (100 μL) were added to each well of the microplate, containing 100 μL of the diluted Blad-oligomer solution (twofold). Final volume in each well was 200 μL. Amphotericin, fluconazole and itraconazole were also tested using the same inocula, but following the dilution scheme described in M38-2A for each case. The microplates were incubated at 25°C without agitation and examined after 72 hours. The MIC endpoints were the lowest drug concentration that showed absence of growth, as recorded visually.

#### Percentage of inhibition of radial growth in agar media

This susceptibility test consists in the assessment of the ability of Blad-oligomer to inhibit the radial growth of fungi in a specific agar medium. The culture medium was selected as the most sensitive for this type of analysis, and it also contained a low concentration of agar, in order to facilitate the diffusion of the oligomer—Potato Dextrose Agar, with 0.6% (w/v) agar and pH 7.5. The oligomer was added to PDA medium (after autoclaving and before pouring in plates) in order to achieve two different final concentrations (4.5 μM and 9.0 μM). The inocula were transferred from the SDA plates using a sterile scalpel blade, by dissecting small agar squares (1–2 mm) containing mycelium and placing them onto the center of each agar plate (one per plate and mycelium facing up). The diameter of the radial growth of the fungus was measured (mm) after 5 days of incubation at 25°C, and the percentage of inhibition of growth was calculated according to the following formula: [(diameter of radial growth in control—diameter of radial growth in medium containing Blad-oligomer)/ diameter of radial growth in control] x 100.

### Field trials

The total 8 days-old cotyledons *Lupinus* extract, containing 0.95 mM of Blad-oligomer, was evaluated for control of fruit rot caused by *Botrytis* spp on strawberries and powdery mildew caused by *Eryshiphe necator* on grapes. Treatments varied among the field trials conducted between 2008 and 2012 (Tables 1 and 2), but common objectives were to determine the field efficacy of Blad-oligomer using full-season treatments. The evaluations were typically conducted close to harvest. In Tables 1 and 2, treatment descriptions indicate the fungicides used, the location of the trial and the rate of active ingredients with spray-intervals according to phenological timmings. As standards-control, common commercially available fungicides were used and are also described in the tables.

**Table 1:** Strawberry fruit rot (*Botrytis cinerea*) field trial treatment description.

Trial	Year	Treatment description^a^	Fungicide^b^/rates	Evaluation^c^
**1**	**2008**	Helena R&D, Watsonville, CA, USA	Non- treated	
		(3 applications@10 day spray intervals)	Blad-oligomer (470g/ha)	**11 April**
			Blad-oligomer (650g/ha	8 DAA3
			Elevate (840 g ai/ha)	
**2**	**2011**	Crop Science, Aromas, CA, USA	Non- treated	
		(5 applications@10–12 day spray intervals)	Blad-oligomer (470g/ha)	**16 September**
			Blad-oligomer (650g/ha	11 DAA5
			Pristine (494g ai/ha)	
**3**	**2011**	Pan Am. R&D, Reno, GA, USA	Non- treated	
		(6 applications@7 day spray intervals)	Blad-oligomer (550g/ha)	**3 June**
			Blad-oligomer (740g/ha	7DAA6
			Endura (450 g ai/ha)	
**4**	**2012**	Hillsboro, OR, USA	Non- treated	
		(5 applications@7 day spray intervals)	Blad-oligomer (450g/ha)	**18 June**
			Blad-oligomer (560g/ha	7DAA5
			Switch (780 g ai/ha)	

^a^ Treatments were replicated 4 times. Plot area was 1 bed (2 arrows of plants) x 9 m. Plots was arranged in a randomized complete block.

^b^ Standards-industrial fungicides use: Elevate—active ingredient: 50% Fenhexamid; Prisitine—active ingredient: 12.8% Pyraclostrobin and 25.2% of Boscalid; Endura—active ingredient: 70% Boscalid; Switch—active ingredient: 37.5% Cyprodinil and 25% Fludioxonil.

^c^ Evaluation for control was made at harvest. 8DAA3, means that the evaluation was made 8 days after application 3.

**Table 2:** Grapevine powdery mildew (*Eryshiphe necator*) field trial treatment description.

Trial	Year	Treatment description^a^	Fungicide^b^/rates	Evaluation^c^
**1**	**2011**	Two bees Agric. Research, CA, USA	Non- treated	
		Carignane	Blad-oligomer (540g/ha)	**13 July**
		(7 applications@10 day spray intervals)	Blad-oligomer (670g/ha	8DAA7
			Pristine (335 g ai/ha)	
**2**	**2012**	Las Condes, Santiago, Argentina	Non- treated	
		Cabernet Sauvignon	Blad-oligomer (335g/ha)	**5 March**
		(4 applications@14 day spray intervals)	Blad-oligomer (450g/ha	14DAA4
			Folicur (170g ai/ha)	
**3**	**2012**	Madera, CA, USA	Non- treated	
		Cabernet Sauvignon	Blad-oligomer (335g/ha)	**2 August**
		(2 applications@21 day spray intervals)	Blad-oligomer (450g/ha	10DAA2
			Prisitne (335 g ai/ha)	
**4**	**2012**	Hillsboro, OR, USA	Non- treated	
		Pinot Noir	Blad-oligomer (335g/ha)	**3 August**
		(6 applications@14 day spray intervals	Blad-oligomer (450g/ha	14DAA6
			Pristine (335 g ai/ha)	

^a^ Treatments were replicated 4 times on 5 vines/plot.

^b^ Standards-industrial fungicides use: Prisitine—active ingredient: 12.8% Pyraclostrobin and 25.2% of Boscalid; Folicur—active ingredient: 20% TEBUCONAZOL.

^c^ Evaluation for control was made at harvest. 8DAA7, means that the evaluation was made 8 days after application 7.

For the strawberry *Botrytis* trials, spraying was made with a backpack spray delivering 1000 L/ha. Plot area was 1 bed (2 arrows of plants) x 9 m. Plots was arranged in a randomized complete block. Incidence was evaluated by harvesting the entire plot and calculating the number of infected fruits per 20 fruits. Severity rating was the average severity of the infected berries sampled in each plot. Treatments were replicated 4 times. For trials number 1, 2 and 3, the disease organism was brought into the trial area and artificially inoculated on strawberries twice during the test period. For the grapevine powdery mildew trials (Table 2), spraying was made with an air blast sprayer with spray direct towards the grape foliage and bunches, delivering 1000 L/ha. Plot area was 28 m^2^, 5 vines per plot, 4 replicates per treatment. The experimental design was a randomised complete block. Incidence was defined as the percentage of grape clusters with powdery mildew within a sample of 20 clusters. Severity was expressed as the percent of berries infected per cluster.

### Oral and contact acute toxicity studies to the honeybee *Apis mellifera* L. of Blad-oligomer

This study was conducted at Eurofins Agrosciense Services, GmbH according to the OECD Guideline No. 213 and No. 214 (1998) [34,35]. The study was carried out with the following treatments in the oral and contact toxicity tests: one dose of the test item (Blad-oligomer); one control in the oral toxicity test (50% w/w sugar solution) and one control in the contact toxicity test (tap water); four doses of the reference item (Perfekthion—a.i. dimethoate). At each dose and treatment, respectively, five replicate groups of 10 bees were tested. For the oral toxicity test, Blad-oligomer was dissolved in tap water to get a stock solution. The test bees were starved for 2 hours before they were fed with the solutions. A quantity of 250 μL of test item or reference item solution was offered to each cage of 10 bees to ensure sufficient consumption of test or reference item. The amount of test solution consumed by each replicate was determined by weighing the feeders (Eppendorf cups) before and after feeding. After a period of 6 h the feeding solution was totally consumed by the bees and the feeders were exchanged. During the observation period the bees were supplied *ad libitum* with untreated 50% aqueous sucrose solution. In the control group the bees were fed with 50% (w/v) aqueous sucrose solution for up to 6 hours after the 2 hours starvation period. For the contact toxicity test, Blad-oligomer was directly dissolved in tap water so that 2 μL contained the required nominal amount of active ingredient per bee. The reference item was dissolved in tap water in order to get a stock solution at the correct concentration for the highest dose so that 2 μL contained the required nominal amount of reference item per bee. After the bees had been anaesthetised with carbon dioxide they were treated individually by topical application with a micro applicator. 2μL of the control, test and reference item solutions were applied dorsally to the thorax of each bee. Between every application, the outside of the micro applicator needle was cleaned with a mixture of water and a water-wetting agent. After application the bees were returned to the test cages and fed with a 50% (w/v) aqueous sucrose solution *ad libitum*. In the oral and contact toxicity tests the number of dead bees in the individual test cages was recorded 4, 24 and 48 hours after the start of feeding and after application, respectively.

### Statistical analysis

In the field trials, the effects of treatments on incidence and severity of both botrytis on strawberry and powdery mildew on grapes were analyzed separately for each trial. A one-way analysis of variance was performed on each dataset, and mean separation (P<0.05) was done by Duncan´s New Multiple Range Test.

In the bee studies, the LD_50_ values with 95% confidence limits of the reference item treatment were calculated by means of a probit analysis using the statistical program SASProprietary Software 9.2, (2002–2008). The oral LD_50_ values for the reference item treatment were calculated with the consumption values per replicate.

### General assays

Protein content was measured using a modification of the Lowry method [36].

The Blad-oligomer content of the 8 days-old cotyledons *Lupinus* extract, was quantified by an HPLC method using as standard a calibration curve made with purified Blad-oligomer.

Affinity-purified, polyclonal anti-ubiquitin antibodies to SDS-treated bovine ubiquitin were produced in rabbits and prepared as described by [37].

## Results and Discussion

### Biochemical characteristics of Blad-oligomer

**Fig 1:**
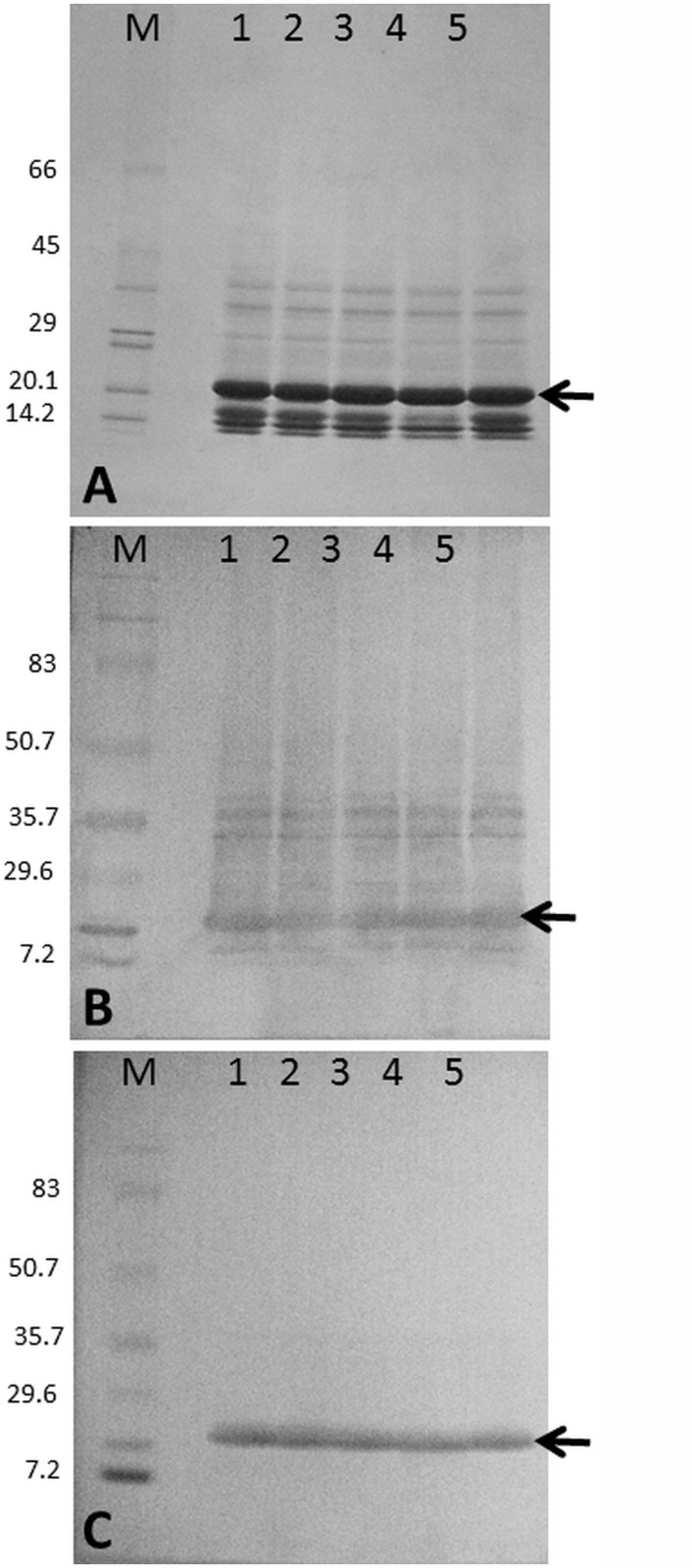
Resistance to inactivation of the lectin activity of Blad-oligomer. Blad-oligomer was purified, incubated for 10 min at 0°C in the presence of water (control, lanes 1 and 5), 4 N HCl (lane 2), 8 N HCl (lane 3) and 12 N HCl (lane 4). The protein samples were subsequently analysed by SDS-PAGE and stained for total protein (50 μg per lane; **A**) or blotted onto a membrane and probed with anti-blad oligomer antibodies (15 μg per lane; **B**) or with affinity purified, anti-ubiquitin antibodies (50 μg per lane; **C**). Molecular masses of standards are indicated in kDa.

The native molecular mass of the native Blad-oligomer has been estimated by gel filtration as 210 kDa. A simple SDS-PAGE analysis reveals that this oligomeric protein is composed of several polypeptides, the major ones exhibiting molecular masses of 14, 17, 20 (Blad; by far, the most abundant), 32, 36, 48 and 50 kDa (Fig 1A, lanes 1 and 5). Previous studies demonstrated that the oligomer is glycosylated, whereas Blad polypeptide is non-glycosylated. Furthermore, among the oligomer, Blad exhibits lectin activity, as evidenced by its ability to bind to antibodies (i.e. immunoglobulins G) and other glycoproteins [38].

**Fig 2:**
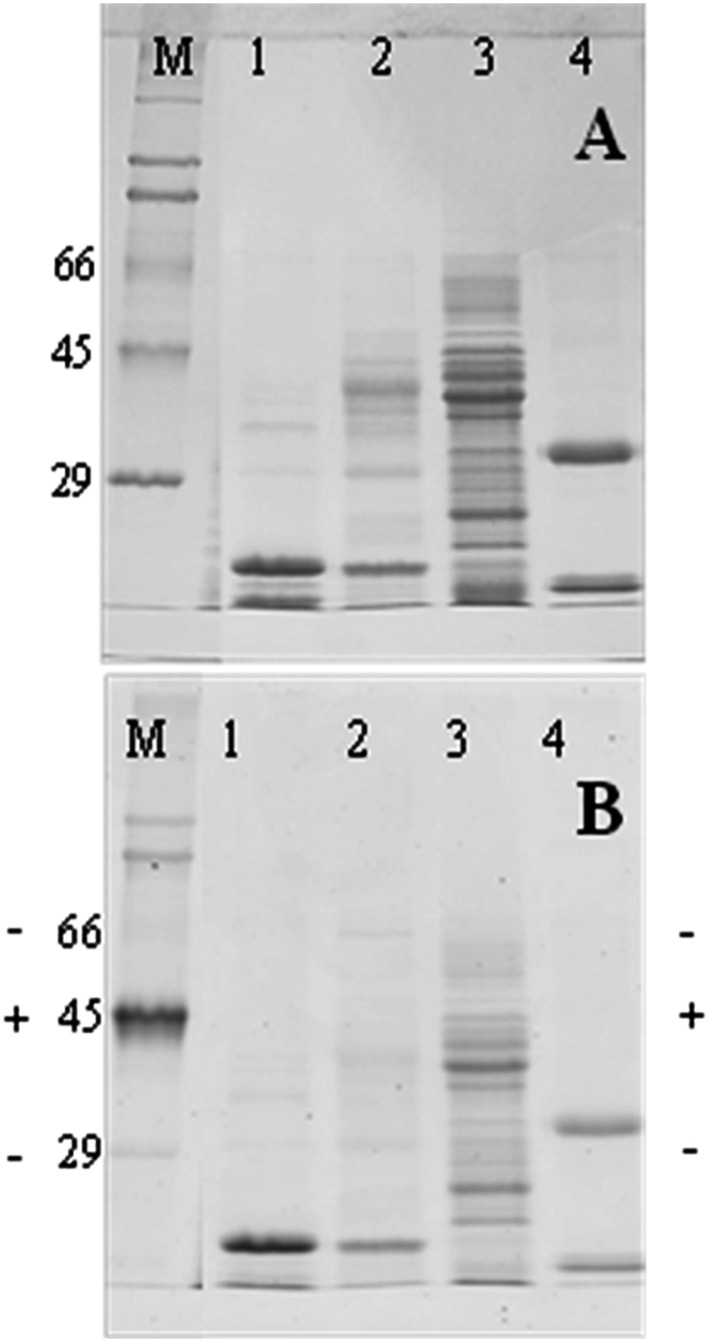
Detection of phosphoryl groups in Blad-oligomer. Blad-oligomer, α, β, and γ-conglutins were purified from L. albus, subjected to SDS-PAGE, and stained for total protein (A) or analysed for the presence of phosphoryl group using the Pro-Q diamond phosphoprotein gel stain (B). Lanes 1, 2, 3, 4: Blad-oligomer, α, β, and γ-conglutins, respectively. Lanes M: molecular mass standards (kDa). The position of the positive (+) and negative (-) phosphorylated markers is shown in B.

When Blad-oligomer, together with the three major storage globulins present in *Lupinus* seeds (i.e. α-, β-, γ-conglutins) were assayed for the presence of phosphoryl groups, the results presented in Fig 2 were obtained. Some of Blad-oligomer sub-units are phosphorylated, as are α-, β- and γ-conglutins sub-units.

The total polypeptides that compose the Blad-oligomer were resolved by SDS-PAGE (Fig 1A, lane 1), transferred onto a blotting membrane and probed with anti-Blad-oligomer antibodies. The results obtained, presented in Fig 1B, lane 1, reveal structural homologies between Blad and some, but not all polypeptides that constitute the oligomer. Probing an identical membrane with affinity-purified anti-ubiquitin antibodies indicates that Blad is the only polypeptide exhibiting lectin activity (Fig 1C, lane 1).

**Fig 3:**
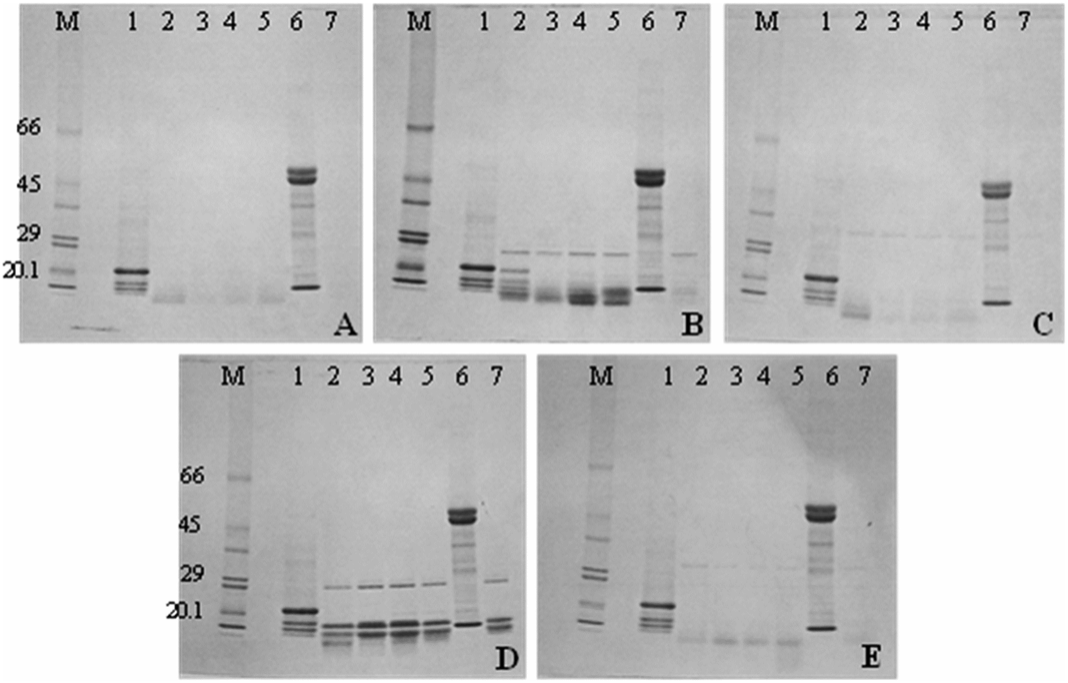
Susceptibility to proteolysis of the Blad-oligomer. Pure 210 kDa protein (lanes 1) was mixed with proteolytic enzymes [pronase in (A); trypsin in (B); proteinase K in (C); α-chymotrypsin in (D); subtilisin in (E)] and incubated at room temperature for 1 h (lanes 2), 2 h (lanes 3) or 2 h followed by addition of pure ribulose bisphosphate carboxylase (55 μg) and a further 1 h incubation (lanes 4). In lanes 5, pure ribulose bisphosphate carboxylase (55 μg) was incubated for 1 h with the corresponding proteolytic enzyme. Lanes 6 and 7 contain pure ribulose bisphosphate carboxylase (55 μg) or the corresponding protease (20 μg), respectively. Lanes M: molecular mass standards (kDa).

The results presented in Fig 1 further indicate that the lectin-like activity of Blad is extremely resistant to inactivation, with the oligomer exhibiting a very high stability against denaturation, withstanding prolonged boiling, treatment with organic solvents and detergents, and exposure to high concentrations of strong acids (e.g. 12 N HCl) as is shown in Fig 1, lanes 2, 3 and 4. In fact, the only treatments which were found capable of abolishing the lectin activity of Blad were those that induce cleavage of peptide bonds [38]. This observation was confirmed by a study on the susceptibility of Blad-oligomer to proteolysis. To this end, the oligomer was mixed with common proteolytic enzymes, namely pronase, trypsin, proteinase K, α-chymotrypsin and subtilisin, and incubated at room temperature for 1 h, 2 h or 3 h followed by addition of a marker protein (55 μg pure ribulose bisphosphate carboxylase) and a further 1 h incubation. Pure ribulose bisphosphte carboxylase was readily degraded by all proteases in all cases. The results obtained after SDS-PAGE analysis of the incubated reaction mixtures (Fig 3) indicate that the Blad-oligomer protein is readily hydrolysed by all proteases tested. This experiment also showed the absence of proteolytic fragments, which could act as proteinase inhibitors or anti-nutritional factors. This conclusion is based on the observation that all proteases tested readily degraded the marker protein added to the reaction medium after the enzymatic digestion of the Blad-oligomer.

### Catalytic activities and chitin affinity of Blad-oligomer

In a subsequent set of experiments, the oligomeric protein was assayed for several enzymatic activities, including those of β-*N*-acetyl-D-glucosaminidase (EC 3.2.1.52), chitinase (EC 3.2.1.14), chitosanase (EC 3.2.1.132), and endo-1,3-β-glucanase (EC 3.2.1.6). Blad-oligomer was extracted and purified from eight days germinated cotyledons and assayed for catalytic activity as described in the Methods section. Two hundred μg of purified protein was used in each assay. All measurements were made in triplicate. The results shown in Table 3 indicate that the Blad-oligomer exhibits high levels of β-*N*-acetyl-D-glucosaminidase (7.38 ± 0.00487 pmol of *p*-nitropheny released per min and μg of protein) and chitosanase (31.9 ± 1.16 pmol of D-glucosamine released per min and μg of protein) activities but no chitinase or β -1,3-glucanase activities.

**Table 3:** Catalytic activities of Blad-oligomer.

Enzymatic activity	Blad-oligomer
β-*N*-Acetyl-D-glucosaminidase	7.38 ± 0.0048^a^
Chitinase	0
Chitosanase	31.9 ± 1.16^b^
Endo-1,3-β-glucanase	0

^a,b^ Catalytic activity is expressed as pmol of *p*-nitrophenyl or D-glucosamine, respectively, released per min μg of protein.

In conclusion, these studies showed that in addition to its role as a seed storage protein, Blad-oligomer binds to immunoglobulins G and other glycoproteins, such as alkaline phosphatase and peroxidase, because it exhibits lectin activity also displaying a very interesting range of enzymatic activities, namely β-*N*-acetyl-D-glucosaminidase and chitosanase activities.

Legume lectin interaction with carbohydrates has long been known to require tightly bound Ca^2+^ and Mg^2+^ (or another transition metal) [39]. A lectin isolated from the bark of *Sophora japonica* has been reported to be self-aggregatable in the presence of Ca^2+^ and Mg^2+^ due to the binding activities of its four subunits, which enable them to recognize and bind *N*-linked oligosaccharide chains on three of the four subunits [40].

As for the *Lupinus* seed storage proteins, including α-, β- and γ-conglutins, Blad-oligomer undergoes a self-aggregation process in a Ca^2+^/Mg^2+^-dependent manner [41]. The simplest explanation would be a self-aggregation of the multivalent lectin activity of the oligomer. However, Ferreira and colleagues [29] provided evidence that this self-aggregation is electrostatic in nature, rather than lectin-mediated. Bridging of calcium ions between negatively charged protein molecules apparently ensures the supramolecular protein association, in the same way as cross-linking between milk submicelles, where calcium ions form bridges between the negatively charged phosphate groups of α- and β-casein molecules present in adjacent submicelles [42]. In this way, the phosphate groups that are present in Blad-oligomer, as well as in α-, β- and γ-conglutins (Fig 2), may participate in this process. Presumably, this self-aggregation mechanism could explain the *in vivo* macromolecular aggregation of legume seed storage proteins that ensures their efficient packing inside the protein storage vacuoles.

**Fig 4:**
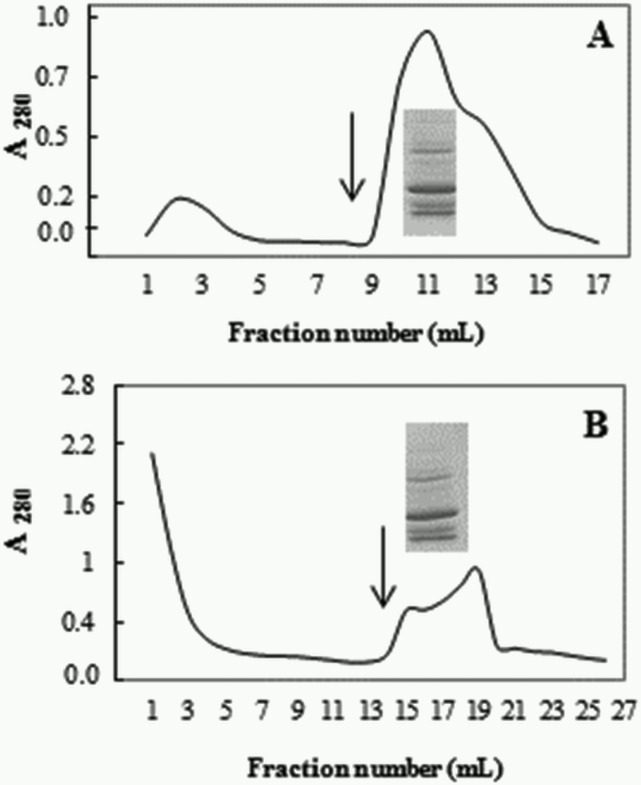
Purification of Blad-oligomer by chitin-affinity chromatography. (A,B) Pure Blad-containing protein (1.2 absorbance units; A) or the total globulin fraction from 8-days germinated *Lupinus* cotyledons (10 absorbance units; B), respectively, were loaded into a chitin column previously equilibrated with 50 mM Tris-HCl buffer, pH 7.5. The column was washed and the bound proteins eluted with 0.05 N HCl (beginning of elution is marked with a vertical arrow). One mL fractions were collected. SDS-PAGE analysis of the polypeptide patterns of Blad-oligomer by the standard, extensive procedure or purified by the single step, chitin-affinity chromatography procedure are illustrated in the figure.

The observations that Blad-oligomer displays a lectin-like activity and catalytic activities of β-*N*-acetyl-D-glucosaminidase and chitosanase prompted us to test whether it also binds to chitin. The results presented in Fig 4 clearly show that the oligomer does bind in a very strong manner to a chitin column, being eluted with 0.05 N HCl. Indeed, this observation led to the development of a single-step purification method to isolate, in a highly purified manner, the native oligomer. The experiment illustrated in Fig 4B shows the single-step, chitin affinity chromatography purification of the Blad-oligomer. The total globulin fraction isolated from the cotyledons of 8-day old *L*. *albus* seeds was directly applied to a chitin column. The column was washed thoroughly and the bound proteins eluted with 0.05 N HCl. The gel shown in the Fig 4 allows a comparison of the polypeptide pattern eluted from the chitin column with that of the Blad-oligomer highly purified by the conventional purification procedure.

Chitin, an obvious natural ligand of Blad-oligomer, has never been detected in higher plants [43]. However, chitin is an important constituent of the exoskeleton arthropods and nematodes and is present in the midgut of some insects as a component of the peritrophic membrane [44]. Chitin is also the major cell wall component in filamentous fungi and a minor constituent in Oomycetes [45]. On the other hand, chitosan is another structural polysaccharide found in fungal cell walls [45]. It is therefore tempting to speculate about the exact physiological role played in *Lupinus* species by the capacity to bind chitin exhibited by Blad-oligomer.

With all this new-coming information available, and regarding that probably the polypeptide Blad, is the main responsible for all this oligomer biochemical characteristic, it becomes increasingly important to work with Blad isolated by conventional biochemical techniques from the other polypeptides that compose the native protein. However, this isolation procedure turned out to be a non-easy task. Besides SDS-PAGE, the only technique that allowed a reasonable isolation of Blad was reverse phase (RP)-HPLC on a C-18 column and even that with a poor level of purification.

### *In vitro* antifungal activity of Blad-oligomer against phytopathogenic fungi

As stated above, besides functioning as a seed storage globulin, Blad-oligomer may well fulfil other physiological roles in the plant. This suggestion is based on the considerable number of distinct biochemical properties exhibited by Blad-oligomer, most notably its catalytic activities, lectin activity and resistance to denaturation. One question naturally emerged at this point of the research—does the oligomer exhibit any anti-biological activity towards those organisms that contain chitin in their structure, namely fungi and insects? To answer this question several experiments were conducted. The susceptibility of several phytopathogenic fungi to the oligomer was assessed *in vitro* by the determination of the Minimum Inhibitory Concentration, i.e., the lowest concentration of Blad-oligomer that inhibits the visible growth of a fungal strain. The same strains were tested against three well-known antifungal compounds (amphotericin, itraconazole, and fluconazole) and the results are shown in Table 4. Blad-oligomer was the only compound that inhibited the growth of all strains tested. The MIC range was relatively narrow, but it is possible to distinguish some species that seem to be more susceptible, namely, *Botrytis cinerea*, *Verticillium* spp. and *Colletotrichum graminicola*. Amphotericin, itraconazole and fluconazole failed to inhibit the complete set of species, although they were tested within the range of concentrations specified in the international standards for this test [33], and MICs were also within the common values for this type of test [46,47]. Although the doses of Blad-oligomer required for fungal inhibition *in vitro* are higher than those usually required for other antifungal drugs, its molecular weight is also substantially higher (210 kDa), which means that the number of molecules required to exhibit the same inhibitory effect is quite similar between all these drugs. Considering the range of MIC values, and expressing the results in number of molecules, the same inhibitory effect is obtained with: 2.04 x 10^13^–2.60 x 10^15^ molecules/mL of amphotericin (MIC μ0.034–4.33 μM), 2.67 x 10^13^–8.53 x 10^14^ molecules/mL of itraconazole (MIC μ0.044–1.42 μM), 1.97 x 10^15^–1.26 x 10^16^ molecules/mL of fluconazole (MIC μ3.27–209 μM), and 8.96 x 10^13^–7.17 x 10^14^ molecules/mL of Blad-oligomer (MIC μ0.15–1.19 μM). Thus, the oligomer seems to have a higher inhibition power by molecule than the azoles and similar to amphotericin. Even if we assume that the polypeptide Blad is the solely molecule responsible for the anti-fungal activity (molecular weight of 20 kDa), the number of molecules required for inhibition is in the range of 9.40 x 10^14^–7.53 x 10^15^ molecules/mL, and is, therefore, quite similar to the values observed for azoles.

**Table 4:** *In vitro* susceptibility of phytopathogenic fungi to Blad-oligomer and other reference antifungal drugs as determined by MIC (Minimum Inhibitory Concentration).

Fungi	MIC (μM)*			
	Amphotericin	Itraconazole	Flucanazole	Blad-oligomer
*Alternaria alternata*	1.08–2.17	0.71	209	0.30–0.60
*Botrytis cinerea*	0.54	0.044	6.53	0.15
*Cercospora zeae-maydis*	0.034	0.35–0.71	3.27–6.53	0.30
*Colletotrichum acutatum*	2.17–4.33	0.71	>209	0.60
*Colletotrichum dematium f*. *trucatum*	4.33	>22.67	>209	1.19
*Colletotricum gloeosporioides*	4.33	1.42	>209	0.60–1.19
*Colletotrichum graminícola*	0.27	0.044	6.53	0.15–62.5
*Fusarium graminearum*	2.17–4.33	>22.67	>209	62.5–0.30
*Fusarium oxysporum*	>17.33	>22.67	>209	0.60
*Verticillium dahliae*	0.135	0.71–1.42	13.06	0.15–0.30
*Verticillium alboatrum*	0.068	0.71	13.06	0.15–0.30

* MIC values were obtained from triplicate analysis and different results between replicates are separated by hyphen.

**Fig 5:**
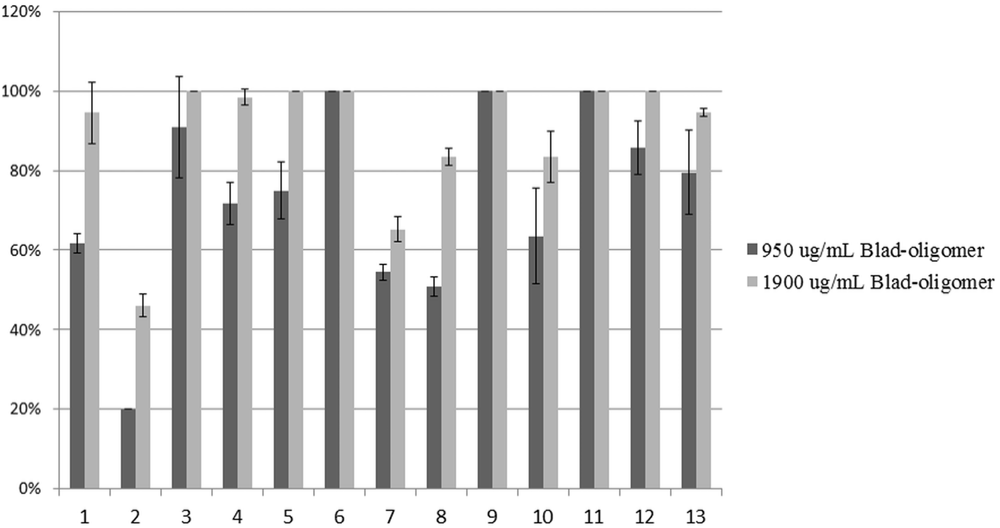
Percentage of inhibition of radial growth of several phytopathogenic fungi in an agar medium containing two different concentrations of Blad-oligomer (4.5 and 9.0 μM). 1: *B*. *cinerea*, 2: *C*. *acutatum*, 3: *C*. *dematium*, 4: *C*. *gloeosporiodes*, 5: *C*. *graminicola*, 6: *E*. *turcicum*, 7: *F*. *oxysporum*, 8: *F*. *solani*, 9: *M*. *fijiensis*, 10: *M*. *phaseolina*, 11: *S*. *sclerotiorum* (CBS 128069), 12: *S*. *sclerotiorum* (CEV—micelium), 13: *S*. *sclerotiorum* (CEV—sclerotia). Values and error bars represent the mean and standard deviation of triplicate measurements.

Another method used to assess the fungal susceptibility to Blad-oligomer was the agar dilution method. This method has the advantage of not requiring a standard spore suspension for inoculum, therefore allowing testing strains which have some difficulties in producing spores in culture medium. Furthermore, it mimics the application of the product in plants, where the contact between the anti-fungal compound and the fungus takes place on the surface of the leaves. However, given the matrix of the agar and the size of the oligomer, diffusion is also more difficult, resulting in a higher dose required for achieving the same inhibitory effect. The results (Fig 5) clearly indicate that growth inhibition was influenced by the concentration of the oligomer, and that there were species particularly susceptible, namely *C*. *dematium*, *E*. *turcicum*, *M*. *fijiensis* and the CBS strain of *S*. *sclerotiorum*. The next most susceptible group, was more dose dependent, and included *B*. *cinerea*, *C*. *gloeosporioides*, *C*. *graminicola*, and the CEV *S*. *sclerotinia*. The less susceptible strains were *C*. *acutatum*, followed by *F*. *solani and F*. *oxysporum*.

As expected from its biochemical properties Blad-oligomer showed a strong antifungal activity. These effects probably result from the strong chitin-binding capacity of the oligomer (Fig 4). Indeed, the antifungal activity of two important families of antifungal proteins, chitin-binding proteins and chitinases, is mainly due to their ability to bind fungal cell wall chitin [4]. Blad-oligomer may therefore be considered as an antifungal compound. Among these, because it is devoid of chitinolytic activity and lacks the chitin-binding hevein domain, it may be classified as a class II chitin binding protein [7,8]. In addition, plant chitin binding proteins have been classified as family 4 pathogenesis-related (PR-4) proteins [8].

Several vicilin-like storage proteins from seeds, including cowpea (*Vigna unguiculata*) and other legume species, were shown to bind to chitin (a β-1,4-linked polysaccharide biohomopolymer of *N*-acetyl-D-glucosamine) and to chitosan (deacetylated chitin) [18]. It has been generally assumed that all chitin-binding proteins for which the amino acid sequence is known contain a common structural motif of 30 to 43 amino acid residues with several cysteines and glycines at conserved positions—this motif is often referred to as the chitin-binding domain [48]. Thus, for example, the vacuolar chitinases of class I possess an N-terminal cysteine-rich domain homologous to hevein and to chitin-binding lectins such as wheat germ agglutinin and *Urtica dioica* lectin [49]. However, extensive sequencing of polypeptide fragments of Blad-oligomer revealed no homologies with chitin-binding domains or the well characterized family of legume lectins, but exhibited an extensive homology with some vicilin-like legume storage proteins, and with α´-subunit of β-conglycinin from *Glycine max* in particular [29]. Interestingly, one of Blad polypeptide internal fragments exhibits high homology with a putative disease resistance protein. The data obtained suggest that the lectin activity associated to Blad-oligomer is unrelated with the so far well characterized legume lectins.

The attack of fungal cell walls by plant chitinases is an important plant defense to fungal infection because it liberates elicitor-active chitin oligomers and weakens the fungal cell wall [50]. However, during invasive growth of hemibiotrophic *Colletotrichum* spp. and biotrophic rust fungi, for example, chitin is exclusively present in the cell walls of exterior infection structures. i.e. germ tubes and appressoria. As a strategy employed by fungi to avoid plant detection and defense, instead of this elicitor-active molecule, the surface of hyphae that grow within host leaves contain chitosan, a deacetylation product of chitin possibly generated by the enzymatic activity of a differentiation induced fungal-chitin deacetylase [51]. The observation that chitosan lacks elicitor activity led Schulze-Lefert and Panstruga [52] to speculate that the “wolf intrudes in sheep’s clothing”. Under these circumstances, the penetration hypha could be protected by enzymatic deacetylation unless the plant tissue possesses an efficient chitosanase activity—Blad-oligomer may well play an important role here, in the protection of *Lupinus* tissues from fungal invasive growth.

### Antifungal activity of Blad-oligomer against grape *Eryshiphe necator* and strawberry *Botrytis cinerea* under field trials conditions

The reasons behind the fact that none of the anti-fungal polypeptides described in literature are being used in the agriculture may be multiple where the sensitivity to the Sun UV radiation can come in first place. At this point the research clearly has been demonstrated that Blad-oligomer has an important activity against plant pathogens in *in vitro* tests. The field conditions are the ultimately step and with utmost importance that could represent the key of success in launching a new natural fungicide to the pesticide market. To address this issue, full-season spray treatments have been performed. Tables 5 and 6 show the results obtained with the oligomer, in the control of fruit rot caused by *Botrytis* spp. (Table 5) on strawberries and Powdery mildew in grapes caused by *Eryshipe necator* (Table 6), when compared with industry-standards fungicides. Due to the fact that *E*. *necator* is an obligate pathogen, *in vitro* studies could not be performed but field trials were included in this study given it economic and environmental burden.

**Table 5:** Strawberry Botrytis fruit rot (*Botrytis cinerea*) incidence and severity in field trials experiments.

Trial	Treatment*	Incidence (%)^§^ on clusters	Severity (%)^§^ on clusters
		**8DAA7** ^ф^	
	Non-treated control	42.0a^¥^	2.0c
**1**	Blad-oligomer (470 g/ha)	27.8a	4.8b
	Blad-oligomer (650 g/ha)	17.5b	3.0c
	Elevate (840g ai/ha)	10.5b	6.8b
		**11DAA5**	
	Non-treated control	46.3a	29.2a
**2**	Blad-oligomer (470 g/ha)	18.8b	11.5b
	Blad-oligomer (650 g/ha)	15.8b	7.1b
	Pristine (494g ai/ha)	16.3b	22.6a
		**7DAA6**	
	Non-treated control	45.0a	38.3a
**3**	Blad-oligomer (550 g/ha)	0.5b	0.13b
	Blad-oligomer (740 g/ha)	0.0b	0.0b
	Endura (450g ai/ha)	0.0b	0.0b
		**7DAA5**	
	Non-treated control	22.0a	17.2a
**4**	Blad-oligomer (450 g/ha)	7.0b	7.0b
	Blad-oligomer (560 g/ha)	4.0b	2.0c
	Switch (780g ai/ha)	5.0b	0.5c

* Treatment conditions are described in Table 1.

^§^ Incidence was evaluated by harvesting the entire plot and calculating the number of infected fruits per 20 fruit. Severity rating was the average severity of the infected berries sampled in each plot.

^ф^ DAA—means days after application.

^¥^ Means followed by the same letter do not significantly differ (P = 0.05, Duncan´s New MRT). Mean comparisons performed only when AOV Treatment P(F) is significant at mean comparison OSL.

**Table 6:** Grapevine Powdery Mildew (*Erysiphe necator*) incidence and severity in field trials experiments.

Trial	Treatment*	Incidence (%)^§^ on clusters	Severity (%)^§^ on clusters
		**8DAA7** ^ф^	
	Non-treated control	92.8a^¥^	21.8b
**1**	Blad-oligomer (540 g/ha)	58.6a	7.8b
	Blad-oligomer (670 g/ha)	24.8b	18.5b
	Pristine (335 g ai/ha)	10.5b	8.8b
		**14DAA4**	
	Non-treated control	50.9aa	39.3a
**2**	Blad-oligomer (335 g/ha)	2.7c	3.3c
	Blad-oligomer (450 g/ha)	5.1c	3.1c
	Folicur (170 g ai/ha)	27.3b	22.6b
		**10DAA2**	
	Non-treated control	100.0a	Non rated
**3**	Blad-oligomer (335 g/ha)	40.4b	Non rated
	Blad-oligomer (450 g/ha)	30.6b	Non rated
	Pristine (335 g ai/ha)	55.2b	Non rated
		**14DAA6**	
	Non-treated control	60.0a	34.5a
**4**	Blad-oligomer (335 g/ha)	3.0b	4.8b
	Blad-oligomer (450 g/ha)	2.3b	1.0b
	Pristine (335 g ai/ha)	2.0b	0.5b

* Treatment conditions are described in Table 2.

^§^ Incidence was defined as the percentage of grape clusters with powdery mildew within a sample of 20 clusters. Severity was expressed as the percent of berries infected per cluster.

^ф^ DAA—means days after application.

^¥^ Means followed by the same letter do not significantly differ (P = 0.05, Duncan´s New MRT). Mean comparisons performed only when AOV Treatment P(F) is significant at mean comparison OSL.

Four trials were conducted in strawberry fruit rot caused by *Botrytis cinerea* (Table 5). In trials number 1, 2 and 3, the disease organism was brought into the trial area and artificially inoculated on strawberries twice during the test period. The disease established well and developed moderate to high infection in the plots. The number of applications differed among the trials and also the interval between sprayings. The rate of disease control was compared with different industrial-standards at a commercial rate (Table 1. Materials and Method section). All plots were rated for *Botrytis* incidence by picking all ripe berries prior to each application. Percent incidence and severity of disease were evaluated. Against a moderate to heavy infestation of *Botrytis* all the treatments provided statistically significant control of *Botrytis* compared to the untreated control. However, the 8-days old *Lupinus* extract at >650 g Blad/ha was the treatment that provided the best control of *Botrytis*, equal or better than the industrial-standards. There was no phytotoxicity to foliage, flowers or fruits from the *Lupinus* extract.

Four Powdery mildew grape trials are reported (Table 6). The number of foliar applications differs among trials but all sprayings were made from pre-bloom trough fruit development. Blad-oligomer was applied at rates between 335–670 g/ha and compared with Pristine at 335 g/ha (trials 1, 3 and 4) or with Folicur at 170 g/ha (trial 2). In trials number 1 and 3, disease was late developing but pressure became intense as the season progressed, with roughly 100% incidence on fruit during July. In all trials both rates of Blad-oligomer as well as the industrial-standards held the powdery mildew infestation and severity of infection to low levels through the rating period. The treated plots for all treatments had significantly less powdery mildew than the untreated control. Treatments with Blad-oligomer were as effective as or better than (P.0.05) the industrial-standards in reducing incidence and severity in the experiments conducted in 2011 and 2012.

### Oral and contact toxicity of Blad-oligomer to the honeybee *Apis mellifera* L.

In a final experiment, due to the nowadays toxicity concerns with fungicides in honeybees population and given the catalytic activities of the oligomer, the toxicity of the Blad-oligomer was tested. The oral and contact toxicity tests were assessed in young adult worker bees derived from a healthy colony which descended from a breeding line of a beekeeper in Rheinland-Pfalz, Germany. Bees were exposed to the oligomer by feeding and by topical application. The test was done during 48 h with 5 replicates, each composed of 10 bees in one cage per test concentration. Mortality was assessed after 4, 24 and 48 hours. In case of symptoms of poisoning, the behavioural differences between the bees of the control group and those of the test item treatment were noted at each observation interval. In the oral toxicity test, the bees of the control group received an aqueous sucrose solution with a final concentration of 500 g/L (50% w/v). In the contact toxicity test, the bees of the control group were treated with tap water. As reference item "Perfekthion" (active ingredient- dimethoate) was used in the oral and contact toxicity test. Four geometrically spaced doses of the reference item were tested.

Table 7 shows the results of the oral toxicity test, which was carried out with the target dose level of 100 μg Blad-oligomer/bee. The actual consumption per bee in the oral test was 109.42 μg Blad-oligomer/bee. The reference item was tested with the nominal dose levels of 0.06, 0.08, 0.11 and 0.15 μg a.i./bee. The mortality in the oral toxicity test is given as a function of the target dose and actual dose of Blad-oligomer or reference item and test solution consumed, respectively. Table 8 shows the results of the contact toxicity test, which was carried out with the target dose level of 100 μg Blad-oligomer/bee. The reference item was tested with the nominal dose levels of 0.10, 0.15, 0.23, 0.34 μg a.i./bee. The LD_50_ values of Blad-oligomer in both tests are presented in Table 9. No mortality occurred in the control groups of the oral and contact toxicity tests during the 48-hour observation period. The 24-hour oral and contact LD_50_ values for the reference item were 0.12 and 0.23 μg dimethoate/bee, respectively. Consequently, validity criteria for both control and reference item mortality were met and the test was deemed valid.

**Table 7:** *Apis mellifera* L. mortality and total consumption in the oral toxicity test in the control, the test item Blad-oligomer and reference item groups.

Treatment (Target dose)	Test item consumed	Mortality [%]	
		24 h	48 h
**Control** (sugar solution)	—	0.0	0.0
**Test item:** Blad-oligomer (μg/bee)			
100	109.42	0.0	0.0
**Reference item:** Perfekthion (μg/bee)			
0.06	0.07	0.0	2.0
0.08	0.09	8.0	18.0
0.11	0.12	62.0	68.0
0.15	0.18	92.0	94.0

**Table 8:** *Apis mellifera* L. mortality in the contact toxicity test in the control, the test item Blad-oligomer and reference item groups.

Treatment	Mortality (%)	
	24 h	48 h
**Control**	0.0	0.0
(Tap water)		
**Test item:** Blad-oligomer (μg/bee)		
100	0.0	0.0
**Reference: item:** Perfekthion (μg ai/bee		
0.10	0.0	0.0
0.15	16.0	26.0
0.23	68.0	72.0
0.34	66.0	66.0

**Table 9:** LD_50_ values in the *Apis mellifera* L. oral and contact toxicity tests with Blad-oligomer.

Blad-oligomer [μg/bee]			
24 h		48 h	
LD_50_	Limits^a^	LD_50_	Limits
**Oral toxicity test**			
> 109.42	-	> 109.42	-
**Contact toxicity test**			
> 100	-	> 100	-

^a^ Lower and upper confidence limits, (95% Confidence interval);

p ≤ 0.05

From a biological point of view, Blad-oligomer may play one or more physiological roles in increasing the chances of plant survival in addition to its function as a storage protein. Indeed, the oligomer appears to reunite in a single molecule selected characteristics of legume seed storage proteins, lectins, antifungal proteins and PR proteins, making it a versatile multifunctional protein—it is a seed storage protein with lectin activity, exhibiting catalytic activities of chitosanase and β-*N*-acetyl-D-glucosaminidase, and the capacity to bind in a strong manner to chitin. It is extremely resistant to chemical inactivation but readily degraded by proteolytic enzymes. These biochemical characteristics explain its intense antifungal properties. Furthermore, the biological properties of the oligomer account for its strange pattern of formation and accumulation in the cotyledons during the germination of *Lupinus* seeds. The oligomer, an intermediate breakdown product of β-conglutin catabolism, abruptly accumulates at about 4 days after the onset of germination, being maintained in high concentrations in the cotyledons during approximately 10 days before being degraded. This pattern of accumulation coincides with the most critical and sensitive phase of the plant development to predation and harsh environmental conditions. Indeed, during the first 4 days of germination, the seedlings are still below the ground and thus naturally protected from most environmental stresses.

The genes encoding many antifungal proteins are currently being used by agribusiness to create genetically modified plants that have increased fungal resistance in the field [53]. The natural susceptibility of proteins to denaturation that results from the typical low free energy required to stabilize mature proteins hampers the direct application of antifungal proteins as fungicide sprays over crop foliages. The exploitation of antifungal proteins extracellularly but in planta or extra planta would require polymers with a reinforced tertiary structure.

From a technological point of view, Blad-oligomer displays a strong antifungal activity, which confers great potential as an antifungal compound for field applications. Its inherent and wide spectrum antifungal activity, initially demonstrated under *in vitro*, is responsible for Blad-oligomer equal or better performance on the control of strawberry *B*. *cinerea* and grapevine *E*. *necator* pathogens under real, open air agricultural conditions, than the best commercial, top chemical fungicides available worldwide. In addition, its extreme resistance to denaturation may allow its use under field conditions; the high susceptibility to proteolytic attack and the specificity of its biological activities probably makes it harmless to the environment and nontoxic to man and animals.

In recent years the decline and disappearance of bee species in the wild and the collapse of honey bee colonies have concerned ecologists and apiculturists, who search for causes and solutions to this problem. Whilst biological factors such as viral diseases, mite and parasite infections are undoubtedly involved, it is also evident that pesticides applied to agricultural crops have a negative impact on bees [54]. LD_50_ results obtained for honeybee toxicity studies clearly points that Blad-oligomer is non-toxic for honeybees being this an imperative requisite for a new active ingredient. Blad-oligomer can be in the future an important alternative for the agriculture around the world.
